# The hidden side of migration: Understanding sexuality as an aspiration to migrate

**DOI:** 10.3389/fsoc.2022.1027268

**Published:** 2023-01-06

**Authors:** Dilvin Dilara Usta, Mustafa F. Ozbilgin

**Affiliations:** ^1^Department of Sociology, University of York, York, United Kingdom; ^2^Brunel Business School, College of Business, Arts and Social Sciences, London, United Kingdom

**Keywords:** migration, sexuality, space, security, endowments, Turkish migrants, UK

## Abstract

Sexuality is an understated yet increasingly important motive for migration. Motivation to migrate is often viewed from a polarized lens, either as the pursuit of economic security or as a desire to access to human rights, on which social policy on migration has been predicated. We introduce the notion of the toxic triangle to account for contexts that prove hostile to freedom of sexuality and trigger individuals to migrate. Drawing on insights from 25 interviews, we demonstrate how sexuality remains a silent yet significant contributor to individuals' decisions to migrate from an adversarial context. We illustrate how the participants fall into four archetypes of dreamers, climbers, escapists, and seekers, based on their endowments and experiences of sexuality and gender identity as salient sources of their motivation to migrate. Focusing on the hidden side of Turkish migration to the UK offers insights into how the pursuit of freedom of sexuality in terms of safety and security shapes motivations and experiences of migration across two cultures.

## Introduction

How could one's sexuality and desire to seek a secure and safe place be related? We expand this question by exploring migrants in the context of their varied capital endowments, i.e., cultural, economic, social and symbolic capital. Contemporary migration studies extensively explore the pursuit of financial, professional, and personal safety and well-being as typical rationales for migration. These studies examine sexuality and gender to understand migrant identities, lives, and experiences (Levitt and Schiller, 2004; Moroşanu, 2018; Engzell and Mathieu, 2020). They suggest migration involves multiple ties and interactions between and within people and national institutions (Janoschka, 2010; Vertovec, 2010). Some scholars have also contextualized migration concerning how the sexuality of migrants interacts with the broader socio-cultural context in which migration occurs (Ahmed, 2000; Fortier, 2001; Mai and King, 2009). Their investigations cover the pursuit of security as a driver for migration, which embeds the aversive state of conflict (Sirkeci, 2009), political risk and engagement (Gamburd, 2000), and the legal safety of migrants (Parreñas, 2001; Pessar and Mahler, 2003). They also indicate that the space in which migration takes place is essential to understanding the migration experience (Luibhid, 2008; Boccagni, 2017; Faist, 2018). We contribute to these studies by exploring sexuality and gender identity as reasons for migration at the interplay of capital endowments of migrants and their struggles for safety and security.

While sexuality and gender identity have been explored by highlighting the implications of migration, studies focused on Turkish migrants only merely engage with broader debates on the intersectionality between sexuality and migration. Over the last decade, Turkish migrants and their reasons for mobility have been broadly studied in migration and globalization studies, specifically in Western Europe (Robins and Aksoy, 2001; Aksoy and Robins, 2003); yet these studies focus on economic and socio-political conventions behind Turkish migration. We contribute to this field by exploring a new set of relational ties by examining the curious relationship between sexuality, security and space in the context of migration.

The paper makes four distinct contributions: First, we illustrate the significant and yet underplayed role that sexuality plays in migrants' decisions to migrate. Second, we show the interplay of sexuality, security, and safety as a context that shapes migrating motivations. Third, we introduce the notion of a toxic triangle to denote a national context that is hostile to expressions of sexuality, which puts individual security at risk and creates danger space for expressions of sexuality. Lastly, we provide a particular approach that goes beyond macro-structural reasons to consider the pursuit of sexual freedom, security and safety for Turkish migration studies. To achieve this, we provide four archetypes of migrants (i.e., dreamers, climbers, escapists, and seekers), each with distinctly different forms of pursuing sexual freedom, by a matrix of space and security dimensions. By doing so, we illustrate how sexuality informs migrants' decisions to migrate from a country/culture of the toxic triangle to the UK.

In the next section, we introduce the notion of a toxic triangle of migration to describe a context where the treatment of sexuality, space and security collude in ways that motivate individuals to migrate to another country. Next, we explain our qualitative data collection method and analysis process. In the findings section, we present four relational archetypes of migrants to illustrate how sexuality forms a significant reason for their migration. In the data analysis and discussion sections, we explore the role of sexuality in migration from a country with a toxic triangle to the UK and investigate the interrelations between sexuality, space and security as reasons for migration in this context.

### The toxic triangle of migration: Sexuality, space and security

We define a toxic migration triangle as a context with hostile structures and cultures against freedom of sexuality. This context presents a danger space for individuals in terms of their freedom of sexuality, which threatens the security of individuals who do not conform to the normative order of sexuality and undermines their symbolic, cultural, social and material endowments. Alternative formulations of the toxic triangle of equality exist in the literature at the interplay of untamed capitalism and human rights (Küskü and Özbilgin, 2021). This paper focuses on migration out of the toxic triangle of sexuality, space and security.

When we chart sexuality and security across space, two distinct contexts emerge: danger space refers to a context in which sexuality is taboo, and legal measures and cultural discourses are ceremonial against harassment and violence (Plummer, 2015; Faist, 2018). The conflict model of migration suggests that danger space triggers individuals to migrate to avoid conflict (Sirkeci and Cohen, 2016). Safe space refers to a context where sexuality can be experienced with freedom and without fear of reprisal (Ayoub and Bauman, 2019; Birgit and Birte, 2020). Although migration has been studied as a generic term, the heterogeneity of migrant lives illustrates that migration is a resource-based phenomenon in which the security of the migrants is tied to their capital endowments (Fouron et al., 2002; Castles et al., 2013; Ferwerda and Gest, 2021). At either end of the spectrum of endowments, i.e., economic, social, cultural and symbolic resources that migrants have, reasons for migration could be varied. Studies on migrants with low levels of capital endowments have highlighted the importance of security, safety, and the pursuit of legal rights as primary reasons for their migration (Ogren, 2007; Van Hear, 2014; Badali, 2019). Whereas migrants with high levels of resources reportedly move for better standards of life, seeking work-life balance, career, escape from past personal and cultural history, and opportunity for self-actualisation, and enjoy higher levels of security in both countries (Benson and O'Reilly, 2009; Castles and Miller, 2009; Salazar, 2010).

Thus, resource-dependent security and safety emerge as salient dimensions that shape migration decisions in the extant literature. Therefore, in this paper, we explore the interplay of sexuality in the context of space and security in shaping motivations to migrate. We frame sexuality in terms of sexual intimacy and gender identity; then, we examine space across safe space and danger space dimensions. We frame security in terms of low and high levels of capital endowments that migrants have. Based on the capital endowments of participants, four archetypes emerge from the data: *dreamers, climbers, seekers*, and *escapists*. The findings section introduces and develops these archetypes and the varied motivations to migrate for each archetype. Below we outline the three aspects of the toxic triangle for migration.

#### Sexuality

Sexuality and gender frame people's opportunities to access economic, legal, social, health, welfare, and education resources. Recent migration studies call for an examination of sexuality within the migration process by highlighting the intersection of sexuality with diverse factors and the question of how sexuality and migration inform and shape each other. This and many other analytical questions related to sexuality and gender have been of significant interest in contemporary migration studies (Manalansan, 2006; Gorman-Murray, 2009, 2019; Mai and King, 2009; Naples, 2020).

Sexuality is often treated in silos, where the studies of heterosexuality and homosexuality have taken divergent paths. At this juncture, non-heterosexual forms of sexuality are often discussed under the banner of LGBTQ+ sexualities and queer theory in migration studies. They reflect the migration experiences of LGBTQ+ migrants by highlighting their belonging, coming-out process, and connection to the community and places (Manalansan, 2006; Gorman-Murray, 2009; Mole, 2018; Luo, 2021). These studies address how LGBTQ+ migrants' migration experiences can be driven by their desire to achieve sexual independence and liberation (Cantú, 2009; Lewis and Naples, 2014). These studies focus on dynamics central to non-heterosexual migrants' migrations.

However, issues related to sexuality and gender began appearing as essential motivations for migration for individuals who are not necessarily sexual minorities. Hence, lately, some queer approaches in migration studies call for examining the full range of sexualities and questioning the normative practices on gender identities beyond heterosexual and homosexual binarism (Mai and King, 2009). Hence, recent studies on sexuality in migration investigate the connections between heteronormativity, sexuality, and migration. These studies' main concern is understanding how non-normative sexuality and gender experiences inform migratory reasons and how they mesh within cross-border socio-cultural and economic structures. These scholars explore the transformation of values and sexualities in the migration process and investigate migration's role in detraditionalising sexual and gender norms (Ahmadi, 2003; Liu-Farrer, 2010; Parrado and Flippen, 2010).

In some cases, sexual and gendered motivations for migration can consist of economic and socio-cultural imperatives. These imperatives are often considered the main trigger for migration; however, migrants' experiences can inform sexual and gendered factors that can cause such imperatives when questioned further. Existing studies acknowledge the economic loss and socio-cultural prosperity in migration, but they underplay the core, decisive role of sexuality within the migration process (Luibhéid, 2015). Thus, in this paper, we consider sexuality as a significant motivation for migration. We also focus on how sexuality shapes global policy (Stonewall, 2021) and migratory processes. We refine the role of sexuality by exploring it in the context of security, i.e., resources that migrants have, and space, i.e., danger and safety, posed by the spaces that migrants occupy.

#### Security: Low and high endowments of capital

Security is often defined as the state's guarantee and assurance to its citizens. From a critical perspective, security is a resource-based phenomenon where individuals depending on the magnitude of their capital endowments, may ensure their security beyond the limited provision of the state. Yet, individuals with low capital endowments would be exposed to higher levels of security risk. Bourdieu defined four different forms of capital endowments: economic, social, cultural, and symbolic capital (Bourdieu, 1986; Ozbilgin and Tatli, 2005; Williams et al., 2020). Economic capital is the endowment of financial and material resources that individuals accumulate. Social capital refers to an individual's endowment of social connections and networks. Cultural capital is an individual's endowments in terms of education, training and experiences, which equip them with expertise, skills, and knowledge (Al Ariss et al., 2013). Individuals are uniquely capable of transposing each capital to another. For example, individuals may use their economic capital to receive cultural capital and use their cultural capital to gain social networks. Symbolic capital is the embodiment of all forms of capital in terms of respectability, recognition and status that an individual accrues. Migrants often experience denigration of their symbolic capital. For example, a medical doctor may find their medical degree unrecognized when they migrate to a country where such recognition of a foreign degree in medicine is not possible. Their financial resources may also be downgraded in purchasing power, and they may lose their social networks. Thus the overall respectability, i.e. their status and respectability as a medical doctor, may be downgraded with migration (Al Ariss et al., 2012; Sayad, 2018).

Security refers to diverse practices that capture different rationales in migration studies, including developing policies to control and surveillance of migrants and frame social transformation as engagements with new places rather than insecurity among borders (Bigo, 2000; Koser, 2005; Rudolph, 2006; Sang et al., 2013), and creating and securing economic, social, cultural and symbolic (freedom of choices and lifestyles) resources and positions of migrants (Benson and O'Reilly, 2009; Rao, 2010; Korpela, 2014; Benson and Osbaldiston, 2016). In this paper, we examined security across the axis of low endowments and high endowments of capital, as groups of migrants experience different security concerns across varying levels of capital endowments.

#### Space: Danger space and safe space

Space provides a specific angle for migration because the interplay of migration experiences and social structures always takes place in a particular space. In the sexuality and migration context, space refers to migrants' sexual and gender experiences and their struggles to find safe spaces. This social space consists of everyday activities, routines, experiences, and social struggles that appear in specific social structures. Hence, social structures inform and shape the social space of migrants that provide knowledge for diverse and oppositional practices, including sexualised, gendered, marginalized, isolated, and danger spaces.

The studies in sexuality and space are primarily ethnocentric, and the experiences of sexual minorities are poorly studied (Binnie and Valentine, 1999). However, focusing on the nexus of sexuality and space reveals a range of conditions where social forces and boundaries shift across spaces (Brown, 2000; Smith, 2012; Lizama, 2019; Kazyak, 2020). As Binnie (1997) notes, 'space is not naturally authentically “straight” but rather actively produced and (hetero) sexualised' (p. 223). Space can be suppressive and permissive to the sexuality and security of migrants. In this sense, migration studies offer two district contexts for space by highlighting the importance of security: danger and safe space. Danger space is oppressive and refers to a context in which sexuality is taboo; security measures are ceremonial against harassment and violence. The conflict model of migration engages with such contexts of danger and conflict that encourage individuals to migrate (Cohen and Sirkeci, 2016). In contrast, a safe space enables the production of diverse sexualized and gendered experiences with freedom and without fear of reprisal (Fortier, 2001; Svašek and Skrbiš, 2007; Lee, 2011; Svašek, 2012; Pilkey et al., 2015; Guha, 2019). In the next section, we explore the toxic interplay between sexuality, security, and space by drawing on the case of the toxic triangle in a country where individuals migrate out to pursue sexual freedom.

### The case of the toxic triangle of migration from Turkey to Western Europe

From the early 1960s to the 1970s, mainland Turkish citizens began to migrate to Western European countries through the guest-worker program of Fordism and bilateral labor agreements between Turkey and European governments (Kirişçi, 2003a,b; Soysal, 2003; Abadan-Unat, 2011; Içduygu, 2011; Pötzschke, 2015). Turkish migration flow from Turkey to Britain demonstrated a wide range of mixtures of high and low endowments. Starting with the 1940s, Turkish migrants presented low endowment qualities with economic and professional resources in their home country; hence their migration was driven by work-related reasons (Robins and Aksoy, 2001) and well as the pursuit of freedom from conflict (Demir and Yazgan, 2019). The current Turkish migration wave displays a high-endowment migrant profile with higher education, professional and economic resources (Ozbilgin and Yildiz, 2022), driven by a complex and divergent set of motives (Sirkeci et al., 2016). A range of reasons has driven their migratory motivation: getting married, getting a job, pursuing higher education, or establishing their own business in the UK. (Pötzschke et al., 2014). Turkish migrants in the UK present both high and low endowments, including those who fled from danger and who left despite having a safe space in Turkey.

Every culture sets norms, rules and expectations about sexuality to provide moral regulations and cultural boundaries to frame forms of sexuality as normal or pathological or acceptable or unacceptable (Plummer, 2015). In Turkish society, cultural norms and religious values play a significant role in understanding sexuality and engagement in sexual acts. Sexuality and gender identity issues are taboo and problematic issues in Turkish society. Women's sexuality is often controlled by religious stricture and cultural values (Ilkkaracan, 2001). The public view of virginity is used to suppress women's sexuality. Awwad (2011) emphasizes that virginity has a moral expression, and honor and shame are the critical elements of virginity controlled in Turkey. According to Human Rights Watch, “more than eleven million women have experienced sexual or domestic violence in Turkey” (Bakshi, 2020). Although Turkey has the highest rate of femicide and trans murders in Europe and one of the highest in the world (Kadin Cinayetlerini Durduracagiz Platformu, 2020), in March 2021, Turkey withdrew from the Council of Europe Convention on preventing and combating violence against women and domestic violence. Turkey presents a perilous setting for women and women's sexuality.

While some countries try to improve the representation of LGBTQ+ individuals in leadership positions (Ozbilgin and Erbil, 2022), LGBTQ+ rights are not recognized, and LGBTQ+ groups are targeted with hate speech, prosecution and persecution by the state authorities in Turkey (Kaos, 2019). Public recognition and legal protection of sexual minorities is a significant problem in contemporary Turkey. Most LGBTQ+ individuals face violence, harassment, stigma and discrimination. Because of social intolerance and lack of legislation regarding the protection of sexual minorities and diversity, many LGBTQ+ individuals stay in the closet and live their lives in fear (Aydın and Ozbilgin, 2019). Indeed, women and LGBTQ+ individuals who do not fit the heteronormative cultural structure of Turkish society face similar changes and struggles. They experience Turkey as a danger space where they endure stigma, social pressure, and a fear of being physically and psychologically harmed. Yet, Ozbilgin (2017) explained that at either end of the capital endowments, individuals in Turkey experience danger and safe space differently. While the wealthier LGBTQ+ individuals have, albeit limited number of venues and levels of social standing and recognition, the lowly resourced, i.e., economically poor, uneducated, working class, and of lower status LGBTQ+ individuals suffer from extremes of danger space. Ozbilgin et al. (2022) also demonstrate how LGBTQ+ individuals across all levels of capital endowment in Turkey are either forced to or choose to pass as cis-gender and heterosexual. Thus, the hostile context for sexuality and lack of security and space for both heterosexual and LGBTQ+ individuals make Turkey an ideal country to study the toxic triangle for migration. Recent figures show that Turkey is now a key source of migration to Europe, particularly to the UK. Sexuality remains an underexplored drive in migrants' motivation to move from Turkey to the UK.

In this sense, we claim that examining the sexuality side of Turkish migration provides significant insights into understanding the connection between sexuality with a wider security system and socio-cultural spaces. More importantly, acknowledging sexuality as a motivation to migrate in Turkish migration studies helps extend studies on reasons to migrate beyond well-established economic and political rationales.

## Method

This paper draws on a qualitative research method that generated in-depth empirical data from fieldwork. Qualitative studies are particularly instrumental for exploring social groups' expressions and experiences of varied cultural and social contexts (Smith and Heshusius, 1986); and empowering researchers to develop analytical frameworks and provide in-depth understandings of hidden factors (Skeggs, 1997). In our study, the qualitative approach allowed us to explore Turkish people's interactions, mutual understanding and negotiations on migration, security and space. The empirical data for this analysis comes from 25 in-depth, semi-structured interviews conducted with Turkish migrants living in the UK in 2018 and 2019.

### Participants

The research was conducted on a sample of Turkish migrants who moved to the UK. Our focus was to examine a wide range of Turkish migrants. We define “Turkish migrant” as someone who has Turkish citizenship, self-identifies as Turkish, Turkish-Kurdish or Kurdish-Turkish, or someone of another ethnicity who migrated to the UK from Turkey. We recruited Turkish migrants with varied endowments with education, professional skills, and socio-cultural security and safety and those who listed sexuality and gender as migratory motivations. Through this, we sought to understand the sexuality-related migration experienced by individuals from diverse backgrounds. Demographic diversity in the sample is an essential element of this research. We interviewed Turkish migrants of various political backgrounds, genders and sexual orientations, cultural backgrounds and religious or secular upbringing. A total of twenty-five Turkish migrants participated in the study. Eleven were Turkish men, of whom five were non-heterosexual, and six were heterosexual. There were fourteen women participants, of whom three were non-heterosexual, and eleven were heterosexual.

### Empirical data: Collection process, interviews, and analysis

The first author of the paper conducted the fieldwork and verbatim transcription of the data. Empirical data were collected through in-depth, face-to-face individual interviews and relational memo-writing for each interview. We adopted a mixture of strategies to recruit potential participants. The research strategies involved inviting eligible participants through the snowballing method, advertising the research details to a member of Turkish societies through gatekeepers and researchers' networks, and posting online research invitations on networks from Turkish migrants' communities. We carefully considered participants' confidentiality and anonymity and used pseudonyms throughout the project.

Participants were invited to talk about their migration experiences, personal and professional desires, sexuality, emotions, and migratory reasons for leaving Turkey. Participants discussed the impact of sexuality and gender on their migration process and highlighted the transformative effect of migration on personal and professional growth. The interviews lasted an average of two hours in a location of the participant's choosing. The interviews were either in Turkish or English, depending on the participant's preference. The interviews were transcribed in both languages until data analysis and interpretation.

We adopted a modified form of grounded theory (Corbin and Strauss, 1990; Charmaz, 2006) and thematic approaches to produce qualitative analysis and data. We encoded the data under three coding stages: initial, focused, and theoretical coding. Considering the limitations of existing studies on sexuality and Turkish migrants, this three-stage coding process enabled us to develop an analytical framework to understand the issues and struggles of Turkish migrants. The final stage of data analysis consisted of a combination of theoretical categories and subcategories with field notes and selecting analysis themes. The thematic analysis allowed us to manage diverse and relational themes and information on systematic levels (Boyatzis, 1998; Guest et al., 2011).

## Findings: Sexuality as a reason for migration for four migrant archetypes

Participants often explained their migration reasons through general socio-economic concerns. Later in the interviews, their desire for security about their sexuality, intimacy, and gender-motivated their initial reasons for migration. Hence, we explore how sexuality, security and space intersect and inform participants' migratory reasons. We focus on how Turkish migrants talk about their migratory motivations based on economic rationales, including socio-economic and education resources, and how these resources increase their security and safety for practicing their sexuality in the host country.

Before we discuss the findings and analysis section, it is essential to explain the archetypes of migrants in terms of security (high and low endowments) and space (danger and safe space). Three core elements emerged from the analysis of participants' narratives: (a) socio-economic and professional status, (b) obtaining a fulfilling life and (c) a desire to increase the freedom of sexuality. Regarding their shared motivations, they fall into two main groups: High Endowment: the dreamers and climbers, and Low Endowment: seekers and escapists. Regarding social space experiences related to sexuality and gender, space implies two dimensions: danger (suppressive) and safe (enabling) space. Four archetypes, including those associated with security and space, are summarized in Table 1 below.

**Table 1:** Four archetypes of migrants by sexuality across space and security.

		**Security**	
		**High endowment**	**Low endowment**
SP	Danger space	Dreamers	Escapists
ACE	Safe space	Climbers	Seekers

### Dreamers (cis-heterosexual women)

*Dreamers* archetype involves participants who are heterosexual women migrants who see themselves as victims of gendered expectations and focus on their agony regarding body, sexual freedom and gender. The migration process provides them with secure space and resources to resolve their dreams and move forward. Although they have high endowments with education, and professional skills, their social space, before migration, was suppressive (danger space) for sexuality.

### Climbers (cis-heterosexual men)

*The climbers* consider and use migration as a catapult to jump or strengthen their heterosexual male privilege in society. They try to reproduce their socio-economic power endowments in a new country through their movement across spaces. Climbers aim for high social prestige and sexual desirability in the UK. They have high endowments with education and a safe space that brings freedom of expression and experiences of sexuality. However, their instability in socio-economic endowments overbalances their self-actualization in stable intimate life. Thus, climbers few express their need to re-stabilize their sexual and socio-economic capital.

### Escapists (sexually marginalized individuals)

*Escapists* seek a safe space to express their authentic sexuality and sexual identity. They are encouraged by their belief in the possibility of having more fulfilling, secure ways of life for sexual minorities like themselves. Their journey refers to their freedom of choice. Their battles with discrimination, inequality and hostility toward their authentic sexuality in their home country contribute to their personal growth and meaning of their life. They have low endowments of socio-economic resources, and they experience danger space regarding freedom of expression and experiences of their sexuality.

### Seekers (sexually marginalized individuals)

*Seekers* pursue essential security and space for their presence in public spaces to achieve their goals. They take more active responsibility to create security and rationales to empower their own needs and ambitions. They negotiate the matter of security and space regarding their needs. They seek opportunities that help to transform their lives.

## High endowment participants

Participants listed a range of factors for their migration. However, they highlighted at least one migratory reason primarily shaped by the search for a sense of security for their sexuality and gender. These reasons are initially driven by their educational, socio-economic background, personal goals, and desires. The data shows how socio-economic and educational reasons for migration were tools for a sense of security in sexuality. For example, getting a higher education, learning a widely spoken language, and having international job experiences and better wages were explained as initial migratory reasons; however, education and socio-economic resources were defined as an occupational concept and a broader opportunity for personal development and sexual explorations.

High endowment participants noted that Turkey's lack of opportunity in securing socio-economic resources created uncomfortable spaces to build secure intimate lives. Some participants changed their perspective through migration, using their socio-economic resources and cultural positions reproduced in the UK (Bourdieu, 1984, 1990). Hence, participants idealized their migratory reasons through their desire to build secure and stable lives and successful careers, to deal with despair in their sexuality, gender identity and lack of intimate practices.

### Dreamers

Gendered expectations in Turkish society bring tensions between constructed womanhood and the process of self-realization in their sexuality and gender identity. Some female participants referred to the asperity of their body, sexuality and gender. Their process of self-realization corresponds to fitting into society and, thereby culturally constructed gender expectations. They believe they fail the general social expectations. Their attachment level with society relates to how they reveal their selflessness. We argue that their motivation to experience their lives in a different society echoes their gendered identity concerns. Their motivations represent a criticism of the desires and expectations of Turkish society. Migration inspires critical thinking about their previous experiences and shows the discovery process of performing their authentic gender and sexuality.

I always felt that I did not meet the cultural expectations of Turkish society… I was just dreaming of being a global person, being able to speak the English language… I was afraid of being sexually harassed because I remember many times I was being followed by strange men and being verbally harassed… I started to think that outside is not safe for women… I wanted to move away from this culture, this view on women… When I turned 20, I came here to live a life I dreamed of and craved. It made me feel like I can finally build my own life, be myself, and choose and experience whatever I wanted. (Güneş, 38, female, heterosexual, full-time professional).

Güneş's reasons to migrate relate to her occupational goals and frame her personal development. She provides a context of cultural and social pressure and safety concerns. She used migration as a strategic tool for her self-discovery process. Her migration narrative displays emotional and sexual intimacy as a reason for mobility. Cicek came with a short-term visa and identified education and occupation as her reasons for migration. Her migrant life brought new sexualised and gendered experiences, reinforcing her decision. She provides a discussion on the transformation of the meaning of mobility and sexual intimacy.

So, I got a two-year visa and found a family to work with… It was a good stable job for two years. I felt empowered after a long time of job struggles in Turkey… When I met my husband, it was a very different relationship. Like we had romance, intimacy like exploration. What we like doing together, activities, sexual bond, maybe this is why we felt confident to marry. I do not think people, especially women, can do these in Turkey. (Cicek, 43, female, heterosexual, full-time professional).

Even if participants accomplish their initial dreams in the UK, they prefer the UK because of its security and safety. Female participants began to perform their authentic gender and sexuality, leading to new sexualised and gender experiences that built reliable intimate lives and marriages. They merged their two diverse life accomplishments (professional and personal) onto the same path. Intimate reasons sustain their dreams of success and integrate their desires with new intimate circles. They think it would be difficult to achieve the same in Turkey. Cicek and others' stories suggest that pursuing sexual intimacy and freedom informed individual strategies to escape the heteronormative structure of gender and sexuality.

### Climbers

Many heterosexual male participants highlighted that stable socio-economic resources secure their manhood in society, which aids them in strengthening their position as a man. Participants' narratives demonstrate the relationship between their sexual and socio-economic capitals and indicate the reflection of manhood in Turkish society.

In Turkey, if you [as a man] do not have a job and career, possibly your ability and knowledge are questioned, and no one wants to associate with you… I could not have a dream, like getting married, or having kids. I could not [bear] those things in there and made decisions… And then [I won the governmental scholarship]. I thought it was an excellent opportunity for me… My main reason is to improve my background for my job, learn a new culture, and have better conditions economically. Now things are better… People stop asking me when I would get a job and how much I earn. They started asking whether I had a girlfriend and when I was going to get married. (Güray, 31, male, heterosexual, full-time professional).

Güray highlighted how having a career, and financial resources are highly valued by Turkish society. His narrative details his gender presentation. Even if Güray's migration initially seemed motivated by economic reasons, his desire for a societal position governed his migratory decision. He believed his lack of financial resources damaged his ‘manhood' and possible intimate life. When he states never imagined himself married in Turkey, his words reveal the interplay between one's economic status and manhood in Turkey.

Existing literature demonstrates that gender traditionalism in Turkey is associated with hetero-patriarchal constitutive norms linked to heterosexual family settings. The impact of religiously inspired sexism and heteronormativity segregates gender roles. That constructive view of gender roles defines a set of standards for the ideal man in society (Levant, 2007). This gender ideology in Turkey classifies men with power, dominance and male gender roles, including fatherhood, breadwinner, and faithfulness (Ugurlu et al., 2018). Men are expected to have high self-esteem and leadership abilities and are socially forbidden to be emotional, approval-seeking, shy or naive. However, there is a hierarchy of power and social status because of limited economic and social resources. Like many others, Güray's story demonstrates the tension at the lower end of this hierarchy.

Moving up the hierarchy is a strategy for many male participants to improve security in their intimate lives. Instead of attempting to move up the social order in Turkey, where economic and social resources are limited, these participants chose to migrate to move up the economic and social hierarchy. Their background assumption is that moving up this economic and social hierarchy makes them more financially and socially attractive and more manly and intimately desirable resultantly. These connections between manhood and power were voiced by participants who questioned how migration could improve and transform their social status in Turkey. These participants explain that cultural and personal factors affect their experiences of manhood and its limited possibilities of sexual freedom in the Turkish context, and migration represents opportunities to expand their repertoires of manhood and male sexuality.

## Low endowment participants

*Seekers* and *escapists* are participants with low endowments of socio-economic and cultural resources to generate authenticity on their sexuality, intimate life and practices and report at least one initial migratory reason related to sexuality and gender. The desire for increased security in sexuality was a more vital migratory reason by participants who are sexual minority individuals and some women. Although initially indicating factors like professional and social development, they reframed those aspirations with security in their sexuality and intimate practices experienced in Turkey. Their narratives demonstrate limited freedom of same-sex intimacy in public space, and insecurities around the female body and sexuality in Turkish society presented salient reasons for migration.

### Escapists

Baha provides the context of how factors for migration are motivated by socio-economic and personal goals and driven by the experience of insecurity in their sexuality in Turkey.

My life and my future are in Europe, not in Turkey, because as a gay man, life is more challenging working in a professional environment... One reason is that if you are a religious minority... gay, or a different sexuality-based minority like me, we do not meet society's expectations. This is why I moved from Turkey even though I love Turkey… I cannot build my future in [Turkey]… I think [Turkey] has become more religious, ignorant, and has less space for the LGBTQ+ community. (Baha, 34, male, gay, full-time professional).

Many other LGBTQ+ individuals experience Baha's challenges as a gay man in Turkish society. Working as a gay academic in Turkey brings many obstacles, such as the risk of losing a job and experiencing discrimination if their sexual orientation is revealed. For some gay participants, migratory reasons originate in economic and cultural mobility, but they admit their sexual orientation and feeling like a minority are salient reasons for their migration. Their feeling of security relates to the ability to build a future as a gay person in society. Migration is driven by occupational and personal goals, which frees them from the danger space of gayness in community and the public. Cengiz explained how gayness and same-sex intimacy have no safe space and security in Turkish society.

It was challenging to accept my sexual orientation in Turkey. I needed to get hefty psychological support. Although I made peace with it, I had to stay in the closet and be hidden from everyone. Because of hostility, discrimination, and social and sometimes individual pressure obligate you to do this. If you would like to live openly, society does not consider you a real man. Hiding and keeping the real you cause deep-level damages… I could not deal with those things anymore. (Cengiz, 26, male, gay, unskilled worker).

Turkish society represents a fragile ground for same-sex intimacy. Cengiz's explanations articulate how social pressure presents the main reason for staying in the closet, causing a dichotomy between a displayed life that meets social expectations and the desired life that engages the authentic self. Cengiz provides evidence of lack of opportunity to experience and express his sexuality. Social bias and sexual discrimination are his main reasons to migrate. His account connects heteronormativity and a low-level tolerance to diverse sexual orientations in Turkey. Life in Turkey provides oppressive space for sexuality for gay men with law capital endowments. Cengiz indicated that migrating to the UK meant leaving a dangerous space. Sexuality and increased safety drove LGBTQ+ participants' migratory reasons. Their concerns about fitting into their home society clash with their inner desires and expectations of how life should be lived. Their journey to the UK symbolizes their freedom of choice. Migration is a strategy to engage with their authentic self and sexuality.

### Seekers

The desire to have a sense of security in sexuality and intimate practices for participants manifests as freedom in public spaces. For example, women's narratives display how female identity and body in the Turkish context are constrained and controlled in public space. However, female participants experienced insecurity while alone in public spaces; they explain sexuality as a factor that encouraged them to leave Turkey.

In the UK you can wear anything, talk about anything, and walk whenever you want. Obviously, I would not go in the night-time on my own, but I can happily go to a pub on my own, I can have a drink on my own, and no one bothers. But in Turkey, especially as a woman, I would not go on my own to a pub because then I would get a label… Violence against women is much higher now than 20 years ago because of political administration in Turkey. This is because they make more male-centered policies and don't tackle all those violence and safety issues… So, those things always made me question what I am doing [in Turkey]. (Meliha, 47, female, heterosexual, full-time professional).

Meliha voices the widespread fears and concerns of women about freedom in public in Turkish society. Their comparisons of dress norms and freedoms in spaces acknowledge Turkey's social and public pressures on the female body. Turkish public spaces inhibit female mobility because of a fear of experiencing social rumors, shame and violence. The social pressure mechanism on female identity works by damaging a woman's dignity. Her experience in the UK empowers her to negotiate her social and cultural position in her homeland.

Similarly, LGBTQ+ participants highlighted issues of expressing sexuality and sexual desire in public settings. Beymen explains how migratory motivation is driven by essential needs for security to express sexual desire and public displays of affection.

I came to establish my own life here [in the UK]. I did believe I could live here under better conditions… like a real human being. At least I would not have to keep a secret and lie to others to be able to live. I had to hide from everybody that I was gay in Turkey… I wanted to show my affections to my boyfriend, walk hand-in-hand, and kiss him on the street… Because of those reasons, I wanted to live in a country where I could experience those things without hesitation. (Beymen, 39, male, gay, unskilled worker).

Beymen shares a fundamental desire to be treated like everyone else. The comfort of expressing his sexuality encourages mobility. Mobility means the freedom to display his sexual identity, desire, and affection to his partner in public spaces. ‘Establishing my own life' represents better and balanced living conditions. The living place dictates a gay man's destiny, so being gay in Turkey means living undercover in many respects. However, living in the UK as a gay man represents coming out of the closet. Beymen's story shows how same-sex couples cannot experience and express their intimacy in public spaces. Public displays of affection were things he had never experienced in Turkey. His sexuality and his need for intimacy manifested as motivations for his migration.

There is a safety issue regarding practicing sexual intimacy in Turkey. Participants' migratory experiences encourage them in two respects: first, through the idea of migration, they begin to critically reconsider their presence and gendered space in their homeland. Awareness of a lack of secure gendered space in Turkey empowers them to look for alternative secure spaces where they can have security for practicing and expressing sexuality. Second, through the migration process, participants develop diverse methods to cope with boundaries created by heteronormative gendered expectations for the female identity and gay identity. Instead of narrowing down their freedom of movement and practicing sexual sexuality in Turkey, they challenge their secure spaces across borders. Hence, their migratory reasons are shaped by their desire to increase safety regarding their sexuality and gender identity.

We outline in Table 2 below how sexuality as a reason for migration manifested across dimensions of space and security from a country of a toxic triangle to a country that afforded migrants more freedom of sexuality. Significantly, the four archetypes in this study had different sexual reasons for migration based on their particular position across material and symbolic safety and their experiences of danger and safe space in Turkey. As outlined in Table 2, our findings indicate that sexuality manifests in different forms as a reason to migrate across space and security.

**Table 2:** Locating sexuality as a migration reason for four archetypes of migrants in terms of sexuality, security, and space.

**Space**	**High endowment (Security)**	**Low endowment (Insecurity)**
Danger space	Dreamers - To break free from socio-sexual expectations, - To bring new gendered experiences in their life	Escapists - To seek safe space and secure endowments to make their life better, - To create security for fitting and focusing on their authentic -self, - To battle with circumstances that force them down the space in which they faced inequality, discrimination and hostility
Safe space	Climbers - To improve fit with gender identity norms - To find a balance between their economic positions and socio-sexual prestige	Seekers - To reclaim body and sexual self-control in public spaces without fear of harm - To pursue self-expression of their sexuality and gender - To break away from limiting cultural norms of intimacy

## Conclusion

The role of sexuality in migration is emerging as a significant yet underplayed phenomenon in migration studies (Mai and King, 2009; Luibhéid, 2015). Further, the interplay between sexuality, security, and space(s) has received little interest in studies on the global human migration process. Our study introduces new ways of thinking and research on human migration by highlighting the underexplored role of sexuality across dimensions of security and space on the decision to migrate. Prior literature on sexuality in migration sought to answer how sexuality and migration shape each other (Luibheid, 2004; Manalansan, 2006; Gorman-Murray, 2009). The previous studies highlighted two approaches for locating sexuality within the migration decision and process. First, they explored sexuality among non-heterosexual migrants to capture their desire and need for sexual freedom and expression (Manalansan, 2006; Cantú, 2009). Second, some scholars have investigated the impact of migration on the detraditionalisation of sexual and gender norms (Ahmadi, 2003; González-López, 2005).

Our study contributes to this body of literature in a new direction by providing empirical evidence that sexuality, desire for freedom of sexual expression and moving away from normative orders of sexuality can be significant migratory motivations for a full range of sexualities. Turkey's toxic triangle of migration significantly underpins all participants' mobility decisions, of which sexuality plays part in different degrees. In this paper, we focus on sexuality, not because it is the dominant reason why these groups of migrants have reported for their migration but because sexuality emerged as a silent yet a significant reason for migration in the field study. For most participants, migration provides secure spaces to explore and practice their sexuality, away from the toxic triangle's cultural and social heritage. Thus, we suggest that sexuality as a reason for migration needs to be explored beyond heterosexual-homosexual binarism and the transformative impact of migration decisions and experience on sexual norms. Yet, we are cautious about significant and nuanced differences in manifestations of sexuality as a reason for migration. To capture some of these fine experiences, we introduced dimensions of safety (low or high capital endowments) and space (danger or safe) that help us frame sexual orientation as a reason to migrate across four different archetypes.

Our research provides important explanations for the interplay of sexuality, security and space that informs migratory motivation. In the literature, security has been studied as a resource-based phenomenon (Ozbilgin and Tatli, 2005; Al Ariss et al., 2013), captured as different rationales (Koser, 2005; Rudolph, 2006; Benson and Osbaldiston, 2016). Studies on security mainly targeted migrants with high endowments and focused on resources and practices to be achieved and secured by this group of migrants. However, in our research, we refined how migrants with low and high endowments of capital experience diverse security-related issues and different priorities of freedom of sexuality. Moreover, our research contributes to migration studies in terms of understanding the social space of migrants formed by specific experiences and struggles related to sexuality. Although migration studies pay particular attention to the nexus between sexuality and migration to understand how social forces and conditions across spaces shape migration (Brown, 2000; Lee, 2011), they provide little explanations on how space can be both suppressive and permissive for security and sexuality of migrants. Hence, we introduce the notion of the toxic triangle of migration to account for the role of sexuality, security and space in shaping migration decisions. Focusing on Turkey, where the repression of sexuality, the lack of security and the experience of danger space constitute a toxic triangle of migration, we reveal sexuality as a significant reason for migration out of Turkey to the UK for migrants across the full spectrum of resource endowments and at different levels of exposure to safety and danger space.

Moreover, we provide a particular approach for future migration studies to go beyond macro-structural reasons to consider human intimacy as a site to study migration. Thus, we suggest that focusing on the economic and human rights rationales and aspects of human intimacy in terms of sexuality and gender identity could enrich and humanize migration studies (Groutsis et al., 2020). Future research can explore the interplay of sexuality as a motive with the dominant rationales of migration, with a view of studying sexuality as a legitimate reason for migration that is worthy of interdisciplinary exploration.

## Data availability statement

The original contributions presented in the study are included in the article/supplementary material, further inquiries can be directed to the corresponding author/s.

## Ethics statement

The studies involving human participants were reviewed and approved by the University of York Management, Politics and Sociology Ethics Committee. The patients/participants provided their written informed consent to participate in this study. Written informed consent was obtained from the individual(s) for the publication of any potentially identifiable images or data included in this article.

## Author contributions

DU was responsible for establishing the theoretical and methodological frameworks of the project, designing, and data collection of the original project and interpreting the findings. MO contributed to data analysis by developing original archetypes of migrants. DU and MO drafted the manuscript and revised the paper. All authors confirmed the final version of the manuscript.
